# [2,7-Dimeth­oxy-8-(2-naphtho­yl)naphthalen-1-yl](naphthalen-2-yl)methanone

**DOI:** 10.1107/S1600536811028054

**Published:** 2011-07-23

**Authors:** Takehiro Tsumuki, Daichi Hijikata, Akiko Okamoto, Hideaki Oike, Noriyuki Yonezawa

**Affiliations:** a Department of Organic and Polymer Materials Chemistry, Tokyo University of Agriculture & Technology, 2-24-16 Naka-machi, Koganei, Tokyo 184-8588, Japan

## Abstract

The mol­ecule of the title compound, C_34_H_24_O_4_, possesses crystallographically imposed twofold *C*_2_ symmetry. The two 2-naphthoyl groups at the 1- and 8-positions of the central naphthalene ring are aligned almost anti­parallel [5.21 (5)°]. The dihedral angle between the central 2,7-dimeth­oxy­naphthalene unit and the terminal naphthyl groups is 75.13 (4)°. In the crystal, weak C—H⋯O hydrogen bonds and π–π stacking inter­actions [centroid–centroid and inter­planar distances are 3.6486 (8) and 3.3734 (5) Å, respectively] are observed.

## Related literature

For the electrophilic aromatic aroylation of 2,7-dimeth­oxy­naphthalene giving aroylated naphthalene compounds, see: Okamoto & Yonezawa (2009 ▶). For the structures of closely related compounds, see: Hijikata *et al.* (2010 ▶); Muto *et al.* (2010 ▶); Nakaema *et al.* (2008 ▶); Nishijima *et al.* (2010 ▶).
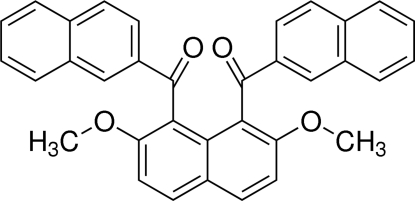


## Experimental

### 

#### Crystal data

C_34_H_24_O_4_
*M* *_r_* = 496.53
Monoclinic, 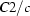

*a* = 12.8325 (5) Å
*b* = 12.2459 (4) Å
*c* = 15.8798 (6) Å
β = 97.618 (1)°
*V* = 2473.41 (16) Å^3^
*Z* = 4
Mo *K*α radiation
μ = 0.09 mm^−1^
*T* = 193 K
0.50 × 0.20 × 0.20 mm

#### Data collection

Rigaku R-AXIS RAPID diffractometer
Absorption correction: numerical (*NUMABS*; Higashi, 1999 ▶) *T*_min_ = 0.958, *T*_max_ = 0.983
19645 measured reflections
2832 independent reflections
2370 reflections with *I* > 2σ(*I*)
*R*_int_ = 0.022

#### Refinement

*R*[*F* ^2^ > 2σ(*F* ^2^)] = 0.037
*wR*(*F* ^2^) = 0.115
*S* = 1.11
2832 reflections
175 parameters
H-atom parameters constrained
Δρ_max_ = 0.28 e Å^−3^
Δρ_min_ = −0.18 e Å^−3^

### 

Data collection: *PROCESS-AUTO* (Rigaku, 1998 ▶); cell refinement: *PROCESS-AUTO*; data reduction: *CrystalStructure* (Rigaku/MSC, 2004 ▶); program(s) used to solve structure: *SIR2004* (Burla *et al.*, 2005 ▶); program(s) used to refine structure: *SHELXL97* (Sheldrick, 2008 ▶); molecular graphics: *ORTEPIII* (Burnett & Johnson, 1996 ▶); software used to prepare material for publication: *SHELXL97*.

## Supplementary Material

Crystal structure: contains datablock(s) I, global. DOI: 10.1107/S1600536811028054/rz2628sup1.cif

Structure factors: contains datablock(s) I. DOI: 10.1107/S1600536811028054/rz2628Isup2.hkl

Supplementary material file. DOI: 10.1107/S1600536811028054/rz2628Isup3.cml

Additional supplementary materials:  crystallographic information; 3D view; checkCIF report

## Figures and Tables

**Table 1:** Hydrogen-bond geometry (Å, °)

*D*—H⋯*A*	*D*—H	H⋯*A*	*D*⋯*A*	*D*—H⋯*A*
C3^i^—H3^i^⋯O1	0.95	2.59	3.3795 (17)	141
C16^ii^—H16^ii^⋯O1	0.95	2.49	3.4382 (14)	175

Symmetry codes: (i) 
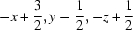
; (ii) 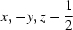.

**Fig. 1.**
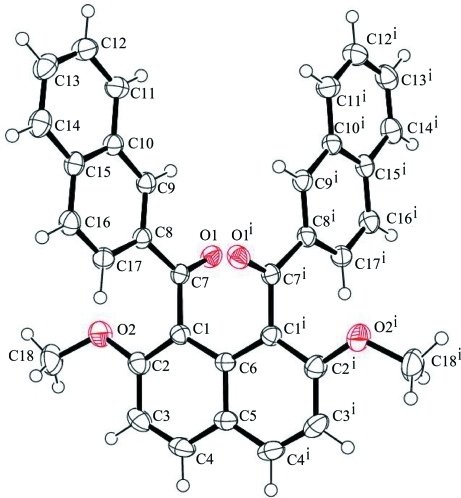
The molecular structure of compound (I). Displacement ellipsoids are drawn at the 50% probability level. Symmetry code: (i) -x+1, y, -z+1/2.

**Fig. 2.**
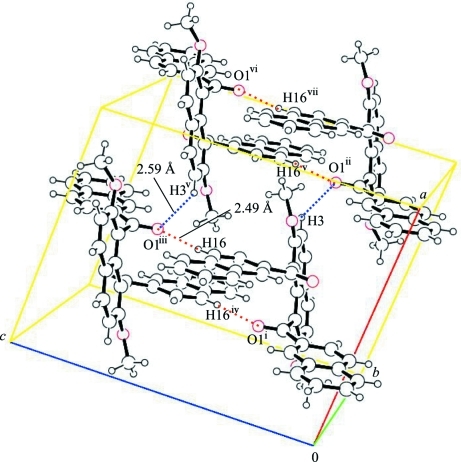
A partial packing diagram of compound (I), showing the C3—H3···O1 (blue dashed lines) and C16—H16···O1 (red dashed lines) hydrogen interactions. Symmetry codes: (i) 1-x, y, 1/2-z; (ii) 3/2-x, 1/2+y 1/2-z; (iii) x, -y, 1/2+z; (iv) 1-x, -y, 1-z; (v) 3/2-x, 1/2-y, 1-z; (vi) 1/2+x, 1/2-y, 1/2+z; (vii) 1/2+x, 1/2+y, z.

**Fig. 3.**
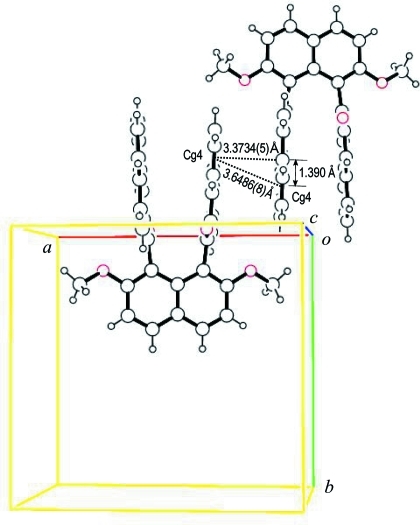
Side view of the π—π stacking interactions (dashed lines). Cg4 is the centroid of the C10–C15 ring.

## supplementary crystallographic information

### Comment

In the course of our study on electrophilic aromatic aroylation of 2,7-dimethoxynaphthalene, *peri*-aroylnaphthalene compounds have been found to be formed regioselectively with the aid of suitable acidic mediators (Okamoto & Yonezawa, 2009). We have reported the X-ray crystal structures of 1,8-diaroylated 2,7-dimethoxynaphthalenes such as 1,8-dibenzoyl-2,7-dimethoxynaphthalene (Nakaema *et al.*, 2008), 1,8-bis(4-methylbenzoyl)-2,7-dimethoxynaphthalene (Muto *et al.*, 2010) and 1,8-bis(4-aminobenzoyl)-2,7-dimethoxynaphthalene (Nishijima *et al.*, 2010). The aromatic rings in these types of molecules are generally assembled with non-coplanar configuration. In these compounds, two aroyl groups tend to attach in nearly perpendicular manner and orient in opposite direction. Recently, the crystal structure of 2,7-dimethoxy-1,8-bis(4-phenoxybenzoyl)naphthalene has been clarified as *syn*-conformation, where two phenoxybenzoyl groups are oriented in the same direction (Hijikata *et al.*, 2010). As a part of our continuous studies on the molecular structures of homologous aroylated 2,7-dimethoxynaphthalene molecules, the X-ray crystal structure of the title compound, (I), bis(2-naphthoylated) 2,7-dimethoxynaphthalene, is discussed in this article.

An *ORTEPIII* (Burnett & Johnson, 1996) plot of the title compound is displayed in Fig. 1. The molecule of (I) lies across a crystallographic 2-fold axis so that the asymmetric unit contains one-half of the molecule. Thus, the two terminal naphthoyl groups are situated in *anti* orientation. The dihedral angle between the central 2,7-dimethoxynaphthalene ring (C1–C6 and C1^i^–C4^i^) and the terminal naphthyl groups (C8–C17) is 75.13 (4)°. The torsion angles of the central 2,7-dimethoxynaphthalene moiety (C1–C6 and C1^i^–C4^i^) and the terminal naphthyl group (C8–C17) with the carbonyl group (C7–O1) are -66.78 (14) [C6—C1—C7—O1] and -179.50 (11)° [O1—C7—C8—C17], respectively.

In the crystal, an oxygen atom of the carbonyl group form two types of intermolecular C—H···O hydrogen bonds with the naphthalene ring hydrogen of the central 2,7-dimethoxynaphthalene moiety [C3—H3···O1 = 2.59 Å] and that of the terminal naphthoyl group [C16—H16···O1 = 2.49 Å], respectively (Table 1 and Fig. 2). Furthermore, an intermolecular π—π stacking interaction is observed between naphthalene rings of the terminal naphthoyl group (C8–C17) with that of the adjacent molecule along the *a* axis [centroid—centroid and interplanar distances are 3.6486 (8) and 3.3734 (5) Å, respectively] (Fig. 3).

### Experimental

To a solution of 2-naphthoyl chloride (629.1 mg, 3.3 mmol) and TiCl_4_ (1802.0 mg, 9.5 mmol) in CH_2_Cl_2_ (2.5 ml), 2,7-dimethoxynaphthalene (188.2 mg, 1.0 mmol) was added. The reaction mixture was stirred at r.t. for 3 h, then poured into ice-cold water (10 ml) and the aqueous layer was extracted with CHCl_3_ (5 ml × 3). The combined organic extracts were washed with 2 *M* aqueous NaOH (20 ml × 3) followed by washing with brine (20 ml × 3). The organic layer was dried over anhydrous MgSO_4_. The solvent was removed under reduced pressure to give a cake (72% yield). The crude product was purified by recrystallization from acetone (22% isolated yield). Furthermore, the isolated product was crystallized from acetone to give single crystals suitable for X-ray amalysis.

Spectroscopic data: ^1^H NMR (300 MHz, CDCl_3_) δ 3.67 (6*H*, s), 7.26 (2*H*, d, J = 9.0 Hz), 7.37 (2*H*, t, J = 7.5 Hz), 7.45 (2*H*, t, J = 7.5 Hz), 7.68–7.77 (8*H*, m), 8.03 (2*H*, d, J = 9.0 Hz), 8.09 (2*H*, brs) p.p.m.; ^13^C NMR (75 MHz, CDCl_3_) δ 56.47, 111.38, 121.73, 124.75, 125.64, 125.92, 127.54, 127.60, 127.83, 129.62, 130.16, 131.11, 132.17, 132.40, 135.50, 136.05, 156.52, 196.66 p.p.m.; IR (KBr):1660(C=O), 1624(Ar), 1510(Ar), 1258(OMe) cm^-1^; HRMS (*m/z*):[*M* + H]^+^ calcd. for C_34_H_25_O_4_, 497.1753; found, 497.1751; m.p. = 505.0–506.0 K

### Refinement

All H atoms were found in a difference Fourier map and were subsequently refined as riding atoms, with C—H = 0.95 (aromatic) and 0.98 Å (methyl), and with *U*_iso_(H) = 1.2*U*_eq_(C).

### Crystal data

**Table:**

C_34_H_24_O_4_	*F*(000) = 1040
*M**_r_* = 496.53	*D*_x_ = 1.333 Mg m^−^^3^
Monoclinic, *C*2/*c*	Melting point = 505.0–506.0 K
Hall symbol: -C 2yc	Mo *K*α radiation, λ = 0.71075 Å
*a* = 12.8325 (5) Å	Cell parameters from 14952 reflections
*b* = 12.2459 (4) Å	θ = 3.2–27.4°
*c* = 15.8798 (6) Å	µ = 0.09 mm^−^^1^
β = 97.618 (1)°	*T* = 193 K
*V* = 2473.41 (16) Å^3^	Block, colorless
*Z* = 4	0.50 × 0.20 × 0.20 mm

### Data collection

**Table:**

Rigaku R-AXIS RAPID diffractometer	2832 independent reflections
Radiation source: rotating anode	2370 reflections with *I* > 2σ(*I*)
graphite	*R*_int_ = 0.022
Detector resolution: 10.00 pixels mm^-1^	θ_max_ = 27.4°, θ_min_ = 3.2°
ω scans	*h* = −16→16
Absorption correction: numerical (*NUMABS*; Higashi, 1999)	*k* = −15→15
*T*_min_ = 0.958, *T*_max_ = 0.983	*l* = −20→20
19645 measured reflections	

### Refinement

**Table:**

Refinement on *F*^2^	Secondary atom site location: difference Fourier map
Least-squares matrix: full	Hydrogen site location: inferred from neighbouring sites
*R*[*F*^2^ > 2σ(*F*^2^)] = 0.037	H-atom parameters constrained
*wR*(*F*^2^) = 0.115	*w* = 1/[σ^2^(*F*_o_^2^) + (0.059*P*)^2^ + 1.0486*P*] where *P* = (*F*_o_^2^ + 2*F*_c_^2^)/3
*S* = 1.11	(Δ/σ)_max_ < 0.001
2832 reflections	Δρ_max_ = 0.28 e Å^−^^3^
175 parameters	Δρ_min_ = −0.18 e Å^−^^3^
0 restraints	Extinction correction: *SHELXL97* (Sheldrick, 2008), Fc^*^=kFc[1+0.001xFc^2^λ^3^/sin(2θ)]^-1/4^
Primary atom site location: structure-invariant direct methods	Extinction coefficient: 0.0039 (6)

### Special details

**Table:**

Geometry. All e.s.d.'s (except the e.s.d. in the dihedral angle between two l.s. planes) are estimated using the full covariance matrix. The cell e.s.d.'s are taken into account individually in the estimation of e.s.d.'s in distances, angles and torsion angles; correlations between e.s.d.'s in cell parameters are only used when they are defined by crystal symmetry. An approximate (isotropic) treatment of cell e.s.d.'s is used for estimating e.s.d.'s involving l.s. planes.
Refinement. Refinement of *F*^2^ against ALL reflections. The weighted *R*-factor *wR* and goodness of fit *S* are based on *F*^2^, conventional *R*-factors *R* are based on *F*, with *F* set to zero for negative *F*^2^. The threshold expression of *F*^2^ > σ(*F*^2^) is used only for calculating *R*-factors(gt) *etc*. and is not relevant to the choice of reflections for refinement. *R*-factors based on *F*^2^ are statistically about twice as large as those based on *F*, and *R*- factors based on ALL data will be even larger.

### Fractional atomic coordinates and isotropic or equivalent isotropic displacement parameters (Å^2^)

**Table:**

	*x*	*y*	*z*	*U*_iso_*/*U*_eq_	
O1	0.61538 (7)	−0.01315 (7)	0.20166 (5)	0.0321 (2)	
O2	0.77075 (7)	0.14496 (8)	0.34570 (7)	0.0453 (3)	
C1	0.59528 (9)	0.14332 (9)	0.28387 (7)	0.0255 (2)	
C2	0.68212 (9)	0.20368 (10)	0.31776 (8)	0.0325 (3)	
C3	0.68048 (11)	0.31884 (11)	0.31852 (9)	0.0389 (3)	
H3	0.7405	0.3590	0.3426	0.047*	
C4	0.59161 (11)	0.37129 (10)	0.28429 (8)	0.0374 (3)	
H4	0.5910	0.4489	0.2834	0.045*	
C5	0.5000	0.31424 (13)	0.2500	0.0299 (3)	
C6	0.5000	0.19760 (12)	0.2500	0.0243 (3)	
C7	0.61088 (8)	0.02168 (9)	0.27324 (7)	0.0245 (2)	
C8	0.61793 (8)	−0.05224 (9)	0.34796 (7)	0.0254 (2)	
C9	0.62457 (9)	−0.16296 (9)	0.33478 (7)	0.0272 (3)	
H9	0.6299	−0.1895	0.2793	0.033*	
C10	0.62360 (9)	−0.23787 (9)	0.40247 (7)	0.0273 (3)	
C11	0.62992 (11)	−0.35233 (10)	0.39025 (8)	0.0351 (3)	
H11	0.6371	−0.3803	0.3355	0.042*	
C12	0.62582 (11)	−0.42292 (11)	0.45670 (9)	0.0401 (3)	
H12	0.6293	−0.4994	0.4476	0.048*	
C13	0.61644 (11)	−0.38257 (11)	0.53837 (9)	0.0392 (3)	
H13	0.6133	−0.4321	0.5840	0.047*	
C14	0.61188 (10)	−0.27273 (12)	0.55253 (8)	0.0360 (3)	
H14	0.6063	−0.2466	0.6081	0.043*	
C15	0.61532 (9)	−0.19709 (10)	0.48511 (7)	0.0281 (3)	
C16	0.61118 (10)	−0.08247 (10)	0.49767 (7)	0.0316 (3)	
H16	0.6079	−0.0544	0.5530	0.038*	
C17	0.61192 (9)	−0.01221 (10)	0.43099 (7)	0.0295 (3)	
H17	0.6084	0.0643	0.4403	0.035*	
C18	0.85102 (12)	0.19798 (14)	0.40216 (10)	0.0513 (4)	
H18A	0.9065	0.1454	0.4217	0.062*	
H18B	0.8208	0.2268	0.4511	0.062*	
H18C	0.8808	0.2581	0.3724	0.062*	

### Atomic displacement parameters (Å^2^)

**Table:**

	*U*^11^	*U*^22^	*U*^33^	*U*^12^	*U*^13^	*U*^23^
O1	0.0388 (5)	0.0320 (4)	0.0255 (4)	0.0067 (4)	0.0041 (3)	0.0003 (3)
O2	0.0253 (5)	0.0440 (5)	0.0631 (6)	−0.0035 (4)	−0.0073 (4)	−0.0025 (5)
C1	0.0250 (5)	0.0252 (5)	0.0265 (5)	−0.0019 (4)	0.0042 (4)	0.0006 (4)
C2	0.0275 (6)	0.0333 (6)	0.0364 (6)	−0.0044 (5)	0.0030 (5)	−0.0011 (5)
C3	0.0376 (7)	0.0343 (7)	0.0442 (7)	−0.0140 (5)	0.0035 (6)	−0.0053 (5)
C4	0.0492 (8)	0.0230 (6)	0.0408 (7)	−0.0078 (5)	0.0087 (6)	−0.0024 (5)
C5	0.0376 (9)	0.0231 (8)	0.0300 (8)	0.000	0.0077 (7)	0.000
C6	0.0280 (8)	0.0216 (7)	0.0238 (7)	0.000	0.0056 (6)	0.000
C7	0.0194 (5)	0.0265 (5)	0.0272 (5)	0.0011 (4)	0.0014 (4)	0.0000 (4)
C8	0.0220 (5)	0.0276 (6)	0.0260 (5)	0.0026 (4)	0.0014 (4)	0.0002 (4)
C9	0.0272 (5)	0.0300 (6)	0.0242 (5)	0.0017 (4)	0.0024 (4)	−0.0018 (4)
C10	0.0244 (5)	0.0278 (6)	0.0292 (6)	0.0008 (4)	0.0016 (4)	0.0010 (4)
C11	0.0415 (7)	0.0289 (6)	0.0346 (6)	−0.0008 (5)	0.0040 (5)	−0.0009 (5)
C12	0.0431 (7)	0.0299 (6)	0.0465 (8)	−0.0007 (5)	0.0026 (6)	0.0058 (5)
C13	0.0375 (7)	0.0413 (7)	0.0383 (7)	−0.0023 (5)	0.0029 (5)	0.0140 (5)
C14	0.0330 (6)	0.0460 (7)	0.0294 (6)	0.0007 (5)	0.0052 (5)	0.0063 (5)
C15	0.0231 (5)	0.0348 (6)	0.0263 (5)	0.0023 (4)	0.0024 (4)	0.0018 (5)
C16	0.0331 (6)	0.0374 (6)	0.0242 (5)	0.0048 (5)	0.0040 (4)	−0.0031 (5)
C17	0.0303 (6)	0.0291 (6)	0.0286 (6)	0.0048 (4)	0.0021 (4)	−0.0037 (5)
C18	0.0336 (7)	0.0641 (10)	0.0521 (8)	−0.0117 (7)	−0.0094 (6)	0.0046 (7)

### Geometric parameters (Å, °)

**Table:**

O1—C7	1.2226 (13)	C9—H9	0.9500
O2—C2	1.3689 (15)	C10—C11	1.4187 (16)
O2—C18	1.4288 (17)	C10—C15	1.4212 (16)
C1—C2	1.3851 (16)	C11—C12	1.3706 (18)
C1—C6	1.4324 (13)	C11—H11	0.9500
C1—C7	1.5154 (15)	C12—C13	1.408 (2)
C2—C3	1.4106 (18)	C12—H12	0.9500
C3—C4	1.358 (2)	C13—C14	1.366 (2)
C3—H3	0.9500	C13—H13	0.9500
C4—C5	1.4131 (15)	C14—C15	1.4209 (17)
C4—H4	0.9500	C14—H14	0.9500
C5—C4^i^	1.4131 (15)	C15—C16	1.4197 (17)
C5—C6	1.428 (2)	C16—C17	1.3653 (17)
C6—C1^i^	1.4324 (13)	C16—H16	0.9500
C7—C8	1.4856 (15)	C17—H17	0.9500
C8—C9	1.3762 (16)	C18—H18A	0.9800
C8—C17	1.4184 (15)	C18—H18B	0.9800
C9—C10	1.4144 (16)	C18—H18C	0.9800
			
C2—O2—C18	117.62 (11)	C9—C10—C15	118.88 (10)
C2—C1—C6	120.06 (11)	C11—C10—C15	119.09 (11)
C2—C1—C7	117.16 (10)	C12—C11—C10	120.61 (12)
C6—C1—C7	122.22 (10)	C12—C11—H11	119.7
O2—C2—C1	115.90 (11)	C10—C11—H11	119.7
O2—C2—C3	122.35 (11)	C11—C12—C13	120.30 (12)
C1—C2—C3	121.65 (11)	C11—C12—H12	119.8
C4—C3—C2	118.83 (11)	C13—C12—H12	119.9
C4—C3—H3	120.6	C14—C13—C12	120.53 (12)
C2—C3—H3	120.6	C14—C13—H13	119.7
C3—C4—C5	122.14 (12)	C12—C13—H13	119.7
C3—C4—H4	118.9	C13—C14—C15	120.76 (12)
C5—C4—H4	118.9	C13—C14—H14	119.6
C4—C5—C4^i^	120.74 (16)	C15—C14—H14	119.6
C4—C5—C6	119.63 (8)	C16—C15—C14	122.22 (11)
C4^i^—C5—C6	119.63 (8)	C16—C15—C10	119.08 (10)
C5—C6—C1^i^	117.65 (7)	C14—C15—C10	118.70 (11)
C5—C6—C1	117.65 (7)	C17—C16—C15	120.65 (11)
C1^i^—C6—C1	124.70 (14)	C17—C16—H16	119.7
O1—C7—C8	121.64 (10)	C15—C16—H16	119.7
O1—C7—C1	118.01 (10)	C16—C17—C8	120.66 (11)
C8—C7—C1	120.33 (9)	C16—C17—H17	119.7
C9—C8—C17	119.60 (10)	C8—C17—H17	119.7
C9—C8—C7	118.51 (10)	O2—C18—H18A	109.5
C17—C8—C7	121.79 (10)	O2—C18—H18B	109.5
C8—C9—C10	121.10 (10)	H18A—C18—H18B	109.5
C8—C9—H9	119.5	O2—C18—H18C	109.5
C10—C9—H9	119.5	H18A—C18—H18C	109.5
C9—C10—C11	122.03 (11)	H18B—C18—H18C	109.5
			
C18—O2—C2—C1	−162.39 (12)	C1—C7—C8—C9	175.44 (10)
C18—O2—C2—C3	21.28 (19)	O1—C7—C8—C17	−179.51 (11)
C6—C1—C2—O2	−177.66 (9)	C1—C7—C8—C17	−0.90 (16)
C7—C1—C2—O2	−6.05 (15)	C17—C8—C9—C10	1.16 (17)
C6—C1—C2—C3	−1.30 (17)	C7—C8—C9—C10	−175.26 (10)
C7—C1—C2—C3	170.30 (11)	C8—C9—C10—C11	179.74 (11)
O2—C2—C3—C4	175.46 (12)	C8—C9—C10—C15	0.17 (17)
C1—C2—C3—C4	−0.7 (2)	C9—C10—C11—C12	−178.16 (12)
C2—C3—C4—C5	1.68 (19)	C15—C10—C11—C12	1.41 (19)
C3—C4—C5—C4^i^	179.28 (14)	C10—C11—C12—C13	−0.7 (2)
C3—C4—C5—C6	−0.72 (14)	C11—C12—C13—C14	−0.3 (2)
C4—C5—C6—C1^i^	178.78 (8)	C12—C13—C14—C15	0.7 (2)
C4^i^—C5—C6—C1^i^	−1.22 (8)	C13—C14—C15—C16	−179.74 (12)
C4—C5—C6—C1	−1.22 (8)	C13—C14—C15—C10	0.01 (18)
C4^i^—C5—C6—C1	178.78 (8)	C9—C10—C15—C16	−1.71 (17)
C2—C1—C6—C5	2.20 (11)	C11—C10—C15—C16	178.71 (11)
C7—C1—C6—C5	−168.97 (7)	C9—C10—C15—C14	178.54 (10)
C2—C1—C6—C1^i^	−177.80 (11)	C11—C10—C15—C14	−1.04 (17)
C7—C1—C6—C1^i^	11.03 (7)	C14—C15—C16—C17	−178.29 (11)
C2—C1—C7—O1	−104.62 (13)	C10—C15—C16—C17	1.97 (17)
C6—C1—C7—O1	66.79 (13)	C15—C16—C17—C8	−0.65 (18)
C2—C1—C7—C8	76.72 (13)	C9—C8—C17—C16	−0.93 (17)
C6—C1—C7—C8	−111.87 (11)	C7—C8—C17—C16	175.37 (10)
O1—C7—C8—C9	−3.17 (16)		

Symmetry codes: (i) −*x*+1, *y*, −*z*+1/2.

### Hydrogen-bond geometry (Å, °)

**Table:**

*D*—H···*A*	*D*—H	H···*A*	*D*···*A*	*D*—H···*A*
C3^ii^—H3^ii^···O1	0.95	2.59	3.3795 (17)	141
C16^iii^—H16^iii^···O1	0.95	2.49	3.4382 (14)	175

Symmetry codes: (ii) −*x*+3/2, *y*−1/2, −*z*+1/2;  (iii) *x*, −*y*, *z*−1/2.
